# Influence of Ionic Liquids on an Iron(III) Catalyzed Three-Component Coupling/Hydroarylation/Dehydrogenation Tandem Reaction

**DOI:** 10.3390/ijms17060860

**Published:** 2016-06-01

**Authors:** Maren Muntzeck, René Wilhelm

**Affiliations:** Department of Chemistry, University of Paderborn, Warburgerstr. 100, Paderborn 33098, Germany; muntzeck@mail.uni-paderborn.de

**Keywords:** ionic liquid, C–H activation, oxidation, iron, catalysis, hydroarylation

## Abstract

A three-component oxidative dehydrogenation tandem reaction via the coupling and hydroarylation of benzaldehyde, aniline and phenylacetylene to a quinoline derivate was catalyzed by an iron-containing ionic liquid. The reaction was air mediated and could be performed under neat conditions. The iron(III) of the ionic liquid was the oxidizing species.

## 1. Introduction

Ionic liquids (ILs), which are salts that have, per definition, a melting point below 100 °C, have attracted much interest in recent years as novel solvents for reactions and electrochemical processes [1]. Several of these liquids can be considered to be “green solvents” [2]. An additional advantage is the efficient recovery of some of these salts. However, for a few examples it is also known that certain ILs are not inert and react with some reagents [3,4,5]. The range of ILs based on various combinations of cations and anions has dramatically increased, and new salts [1,6,7,8,9,10,11,12,13] are continuously being reported. In the 1960s ILs were prepared from organic cations and chloroaluminate anions [14]. Since AlCl_3_ was present in these liquids, the latter were also used as catalysts in Lewis acid–catalyzed reactions. In addition, many of the ILs developed in the 1990s, which incorporated relatively inert lipophilic anions, have been used as solvents for catalytic reactions [15,16,17,18,19]. However, it is also known that these ILs are capable of catalyzing reactions, either in substoichiometric amounts or as reaction media due to stronger interactions with the reaction partners compared to some standard solvents [20].

Next to ionic liquids incorporating chloroaluminate anions, other metal-containing ionic liquids are known [21]. Especially metallic ionic liquids such as [BMIM][FeCl_4_] seem to have good properties for oxidative reactions. This type of ionic liquid can work as a Lewis acid and as an oxidant at the same time. There are examples known where [BMIM]Cl in combination with FeCl_3_ is successfully used for the oxidative desulfurization of fuels [22,23]. With [FeCl_4_]^−^-based ionic liquids oxidations of organic halides to aldehydes and ketones were also performed. The IL was used in substoichiometric amounts with H_5_IO_6_ as the oxidation agent [24]. However, tetrachloroferrate-based ionic liquids have been mainly used in reactions as Lewis acidic solvents [25].

Due to our interest in ionic liquids [4,26,27,28,29,30,31,32,33,34], we wanted to explore the catalytic oxidative behavior of tetrachloroferrate-based ionic liquids in the synthesis of a quinoline derivate. Quinoline derivates and quinoline itself are important structural units in a variety of biologically active natural products. They are also necessary starting materials for the chemical and pharmaceutical industry [35,36,37]. One of the most common methods for creating quinolines is the Friedländer reaction. It is based on an aldol condensation of 2-aminobenzaldehydes. Due to the instability of 2-aminobenzaldehydes, these are generated *in situ* by the reduction of 2-nitrobenzaldehyde derivatives, which are not readily available [38,39,40,41]. Therefore, the development of quinoline syntheses has attracted increasing attention and has been studied by several groups over the last years. Especially synthetic methods based on the use of transition metal catalysts showed good results as they can give the desired products in a simple one-step reaction with easily available starting materials [42,43,44,45,46,47]. Furthermore, there are some examples of the synthesis of quinolines using different iron salt–based catalysts [48,49,50]. Iron salts are a promising catalyst for a variety of reactions and iron is outstanding due to its non-toxicity, low price and environmentally friendly features [51].

## 2. Results

We chose the reaction of benzaldehyde **1** with aniline **2** and phenylacetylene **3** to 2,4-diphenylquinoline **4** as the model reaction. The results are summarized in Table 1. First only FeCl_3_·6H_2_O was applied as the catalyst under an atmosphere of air in toluene. The reaction time was 24 h and the reaction temperature was 110 °C.

**Scheme 1:**
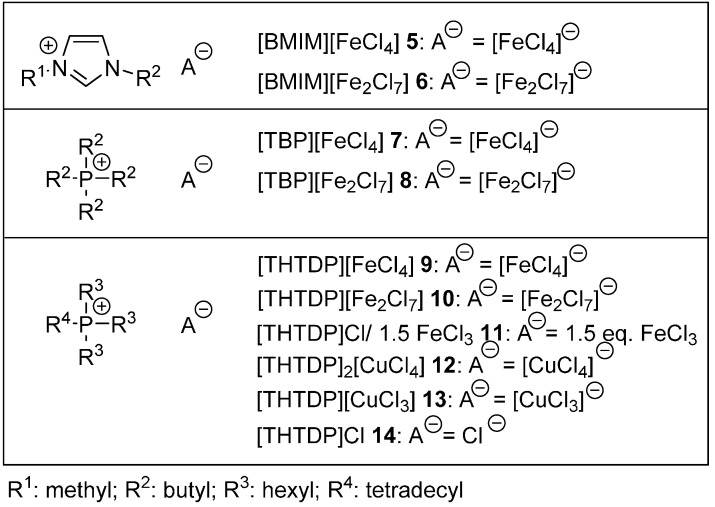
Structures of metallic ionic liquids.

The hexahydrate salt of iron(III) chloride was chosen since many ionic liquids are known to be hygroscopic and contain some amounts of water [1]. These conditions gave a baseline for the further reactions. Next, several ionic liquids were applied in the catalytic reaction. The used ionic liquids, depicted in Scheme 1, were 1-butyl-3-methylimidazolium tetrachloroferrate [BMIM][FeCl_4_] **5**, 1-butyl-3-methylimidazolium heptachlorodiferrate [BMIM][Fe_2_Cl_7_] **6**, tetrabutylphosphonium tetrachloroferrate [TBP][FeCl_4_] **7**, tetrabutylphosphonium heptachlorodiferrate [TBP][Fe_2_Cl_7_] **8**, trihexyl(tetradecyl)phosphonium tetrachloroferrate [THTDP][FeCl_4_] **9**, trihexyl(tetradecyl)phosphonium heptachlorodiferrate [THTDP][Fe_2_Cl_7_] **10**, trihexyl(tetradecyl)phosphonium chloride with 1.5 eq. FeCl_3_ **11**, bis-trihexyl(tetradecyl)phosphonium tetrachlorocuprate [THTDP]_2_[CuCl_4_] **12**, trihexyl(tetradecyl)phosphonium trichlorocuprate [THTDP][CuCl_3_] **13** and trihexyl(tetradecyl)phosphonium chloride [THTDP]Cl **14**.

The focus of the presented work was on ILs with [FeCl_4_] as the anion; however, two copper-based ILs were also explored. First, ILs with an anion-to-cation ratio of 1:1 were applied. None of these ILs **5**–**7** (Table 1, entries 2–4) supported the reaction. Hence, the metal content was changed to a ratio of 2:1 (FeCl_3_ to IL). The best result was achieved by using [TBP][Fe_2_Cl_7_] **9** which gave the product in 62% yield (Table 1, entry 8). The other ILs with a higher iron content gave yields of 53% ([THTDP][Fe_2_Cl_7_] **10**) and 44% ([BMIM][Fe_2_Cl_7_] **8**) (Table 1, entries 5 and 9). Neat reaction conditions at 130 °C with compound **8** as the catalyst resulted in a low yield of 16% (Table 1, entry 7).

For the further experiments, the focus was directed on the reaction with [THTDP] iron salts as catalysts, due to their low viscosity. With [THTDP][Fe_2_Cl_7_] **10** and 1,2-dichloroethane as the solvent at 85 °C, a yield of 50% was obtained (Table 1, entry 10). Under neat reaction conditions with [THTDP][Fe_2_Cl_7_] **10** as the catalyst, a yield of 30% was found (Table 1, entry 11). The use of a higher reaction temperature at 130 °C in combination with neat conditions raised the yield to 38% (Table 1, entry 12). Next, the system was investigated by performing the reaction in a microwave oven. Under neat reaction conditions and 110 °C for 10 min (150 W), a yield of 41% was obtained (Table 1, entry 13). With the achieved results so far, it was concluded that the ratio of the metal ion to the cation has an important influence. Therefore, another ionic liquid with an anion-to-cation ratio in between the two investigated ranges was applied in the reaction. This ionic liquid consisted of 1.5 equivalents iron trichloride to one equivalent [THTDP]Cl **14**. The reaction was performed in toluene and 1,2-dichloroethane. In toluene at 110 °C, a yield of 48% was obtained while the reaction in 1,2-dichloroethane at 85 °C gave a yield of 38% (Table 1, entries 14–15). The change of the reaction time to 14 h in toluene lowered the yield to 38% (Table 1, entry 16). Under neat conditions, the yield of the reaction was 36% (Table 1, entry 17). Raising the temperature to 130 °C under neat conditions did not improve the yield (31%) (Table 1, entry 18). Under microwave conditions, a yield of 10% was obtained after 10 min (Table 1, entry 20). Prolonging the reaction time to 20 min changed the yield to 43% (Table 1, entry 19). In addition, an experiment was performed with the metal-free IL [THTDP]Cl **14**. As expected, the reaction failed in the absence of iron (Table 1, entry 21). Furthermore, the reaction was investigated with copper salts; however, no desired product could be isolated (Table 1, entries 22–24).

## 3. Discussion

**Scheme 2:**
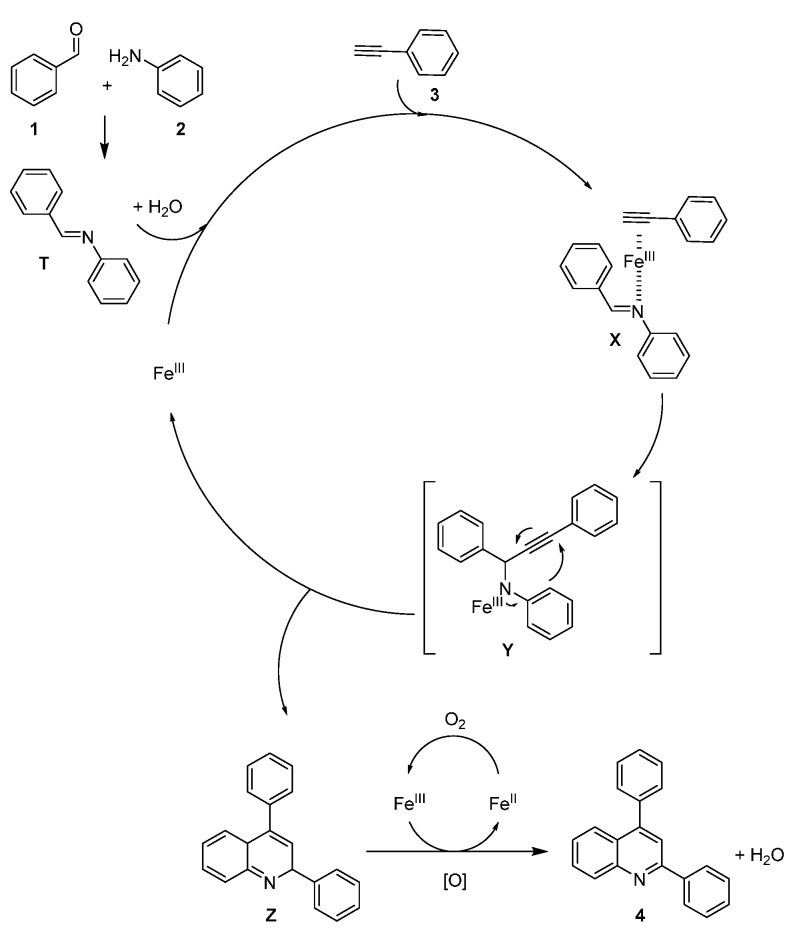
Possible mechanism for the oxidative tandem and hydroarylation reaction of benzaldehyde **1**, aniline **2** and phenylacetylene **3**.

The examined reaction depends on the transition metal catalyst. No reactions were observed when copper catalysts were tested in place of iron analogs. The iron(III) functioned as a Lewis acid and as an oxidant. Taking the result with iron(III) chloride hexahydrate and the literature results by Tu *et al.* [48] into account, it can be concluded that the reaction’s performance in the presence of water is not as good as in the absence of water. Ionic liquids are known to always contain a certain amount of water so the reaction yield may be lowered by this fact [1]. However, due to the fact that the first step of the reaction is the formation of an imine species **T** (Scheme 2), water is formed during the reaction. The imine was present after the reaction and was also isolated; hence, the imine formation was not the limiting parameter in the reaction. A yield of 53% was achieved when iron(III) chloride hexahydrate was used under an atmosphere of air. By using [TBP][Fe_2_Cl_7_] **9** the yield was increased to 62%. The three different cations investigated differed strongly in their structure. The [BMIM] cations are known to change to less stable mesomeric structures that can build complexes with metalates [52,53]. Furthermore, [BMIM] cations are less stable because of this change in their structure and are more likely to react under harsh reaction conditions. In addition, [BMIM] cations can be activated at the C(2)–H bond. The other two cations applied differ in their chain length. Both cations contain phosphorus, but the [TBP] cation has shorter chains and is more symmetrical than the [THTDP] cation. Therefore, [TBP][Fe_2_Cl_7_] **9** is less lipophilic than [THTDP][Fe_2_Cl_7_] **10**.

**Scheme 3:**
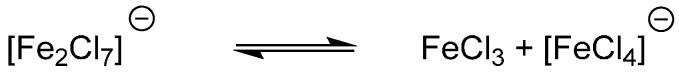
Equilibrium of the [Fe_2_Cl_7_] anion.

The reaction did not give the desired product when the ionic liquids were used with FeCl_3_ in a ratio of 1:1. This can be explained considering the possible reaction mechanism. A tentative mechanism for the reaction is shown in Scheme 2 [48]. As mentioned before, in the first step benzaldehyde **1** forms with aniline **2** an imine **T** *in situ* through a condensation reaction. The Lewis acidic iron(III) coordinates with this imine **T** and phenylacetylene **3** to form intermediate **X**. The propargylamine **Y** is formed through the addition of acetylene **3** to the imine **T**. It forms the dihydroquinoline intermediate **Z** by performing an intermolecular hydroarylation. **Z** is oxidized by iron(III) which is reduced to iron(II) in this process. The iron(III) species is regenerated by oxygen in the air atmosphere. This mechanism is based on the equilibrium of the [Fe_2_Cl_7_] anion, which is shown in Scheme 3.

As can be seen in Scheme 2, iron(III) is acting as a Lewis acid and coordinates the reagents. At the same time, an iron(III) species is used as an oxidant. Furthermore, free FeCl_3_ with an accessible coordination side is necessary. Therefore, an excess of iron trichloride is needed. This theory is based on the experiments with [THTDP]/1.5 FeCl_3_ as a catalyst, which gave lower yields than [THTDP][Fe_2_Cl_7_] **10**. It is possible that in the oxidation step, iron(III) is the oxidant. It is also possible that oxygen from the air is acting directly as the oxidant. We base our first assumption on the fact that the reaction solution turns from a yellow shade that is most likely from the iron(III) salt to a green color which is characteristic of iron(II) salts. The ionic liquid changes its color back to yellow when it is stored in air. Through this, it will be possible to reuse the ionic liquid for further reactions. The fact that [FeCl_4_]^−^-based ILs gave no yields whatsoever can be attributed to the fact that an equilibrium between FeCl_3_ + [Cl] and tetrachloroferrate is far on the latter side. Hence, FeCl_3_ is not present in sufficient amounts with a free coordination site on the iron(III) center.

## 4. Materials and Methods

### 4.1. General Methods

The [BMIM][FeCl_4_] **5** [54] and [BMIM][Fe_2_Cl_7_] **6** [55] were prepared according to the literature from [BMIM]Cl which was purchased from commercial sources. The [TBP][FeCl_4_] **7** [56] and [TBP][Fe_2_Cl_7_] **8** were prepared according to the literature from [TBP]Cl which was purchased from commercial sources. The [THTDP][FeCl_4_] **9** [57], [THTDP][Fe_2_Cl_7_] **10**, [THTDP]Cl/1.5 FeCl_3_ **11**, [THTDP]_2_[CuCl_4_] **12** and [THTDP][CuCl_3_] **13** were prepared according to literature from [THTDP]Cl which was purchased from commercial sources. All of the other reagents were commercially available and used as supplied without further purification. Solvents were either employed as purchased or dried according to procedures described in the literature. IR spectra were recorded on a Bruker VERTEX 70 FT-IR spectrometer. The ^1^H-NMR and ^13^C-NMR spectra were recorded at 500 MHz or 125 MHz on a Bruker Avance 500 spectrometer using the deuterated solvent as the lock and the residual solvent or TMS as the internal reference. Mass spectra were obtained on a Bruker Esquire 3000 plus mass spectrometer (Bruker-Franzen Analytik GmbH, Bremen, Germany) equipped with an ESI interface and an ion trap analyzer. High-resolution mass spectra (ESI) were recorded on a Waters Quadrupole-ToF Synapt 2G. The reactions were followed by thin layer chromatography on silica gel precoated plates (Merck TLC silica gel F254). Column chromatography was performed using silica gel 60.

### 4.2. General Experimental for the Preparation of 2,4-Diphenylquinoline **4**

Benzaldehyde **1** (106 mg, 0.101 mL, 1.0 mmol), aniline **2** (98 mg, 0.096 mL, 1.05 mmol), phenylacetylene **3** (153 mg, 0.165 mL, 1.5 mmol) and [THTDP][Fe_2_Cl_7_] **10** (83 mg, 0.1 mmol) were added to a 10 mL flask under an atmosphere of air. After toluene (1 mL) was added, the reaction mixture was stirred at 110 °C for 24 h. Afterwards the reaction mixture was cooled to room temperature. The purification was performed by flash chromatography (petroleum ether/AcOEt = 20:1) to afford **4** (136 mg, 50% yield). ^1^H-NMR(CDCl_3_, 500 MHz, ppm): δ 8.31–8.29 (d, *J* = 10 Hz, 1 H), 8.25–8.24 (m, 2 H), 7.95–7.93 (d, *J* = 10 Hz, 1 H), 7.86 (s, 1 H), 7.78–7.75 (m, 1 H), 7.60–7.48 (m, 9 H); ^13^C-NMR(CDCl_3_, 125 MHz, ppm): δ 156.8, 149.1, 148.8, 139.6, 138.4, 130.1, 129.5, 129.4, 129.3, 128.8, 128.5, 128.4, 127.6, 126.3, 125.8, 125.6, 119.3 (Figure S1). The spectral data of the product was consistent with literature values [48].

### 4.3. Preparation of Metallic Ionic Liquids

1-Butyl-3-methylimidazolium tetrachloroferrate [BMIM][FeCl_4_] **5**: [BMIM]Cl (1 g, 5.8 mmol) and FeCl_3_·6H_2_O (1.56 g, 5.8 mmol) were stirred at RT for 30 min in a 10 mL round bottom flask. The developing water phase was separated and the ionic liquid was dried for three days under vacuum. [BMIM][FeCl_4_] **5** was obtained as viscous brown oil (1.95 g, 5.8 mmol, 100%).

1-Butyl-3-methylimidazolium heptachlorodiferrate [BMIM][Fe_2_Cl_7_] **6**: [BMIM]Cl (0.5 g, 2.9 mmol) and FeCl_3_·6H_2_O (1.56 g, 5.8 mmol) were stirred at RT for 30 min in a 10 mL round bottom flask. The developing water phase was separated and the ionic liquid was dried for three days under vacuum. [BMIM][Fe_2_Cl_7_] **6** was obtained as viscous brown oil (1.45 g, 2.9 mmol, 100%).

Tetrabutylphosphonium tetrachloroferrate [TBP][FeCl_4_] **7**: [TBP]Cl (1.7 g, 5.8 mmol) and FeCl_3_·6H_2_O (1.56 g, 5.8 mmol) were stirred at RT for 30 min in a 10 mL round bottom flask. The developing water phase was separated and the ionic liquid was dried for three days under vacuum. [TBP][FeCl_4_] **7** was obtained as yellow powder (2.65 g, 5.8 mmol, 100%). IR: ν (cm^−1^): 2963, 2931, 2876, 1464, 1404, 1381, 1231, 1188, 1100, 1006, 968, 918, 812, 713 (Figure S2).

Tetrabutylphosphonium heptachlorodiferrate [TBP][Fe_2_Cl_7_] **8**: [TBP]Cl (0.85 g, 2.9 mmol) and FeCl_3_·6H_2_O (1.56 g, 5.8 mmol) were stirred at RT for 30 min in a 10 mL round bottom flask. The developing water phase was separated and the ionic liquid was dried for three days under vacuum. [TBP][Fe_2_Cl_7_] **8** was obtained as yellow powder (1.8 g, 2.9 mmol, 100%). IR: ν (cm^−1^): 2964, 2932, 2876, 1464, 1404, 1381, 1229, 1188, 1100, 1004, 969, 918, 812, 713 (Figure S3).

Trihexyl(tetradecyl)phosphonium tetrachloroferrate [THTDP][FeCl_4_] **9**: [THTDP]Cl (3 g, 5.8 mmol) and FeCl_3_·6H_2_O (1.56 g, 5.8 mmol) were stirred at RT for 30 min in a 10 mL round bottom flask. The developing water phase was separated and the ionic liquid was dried for three days under vacuum. [THTDP][FeCl_4_] **9** was obtained as yellow oil (3.95 g, 5.8 mmol, 100%). IR (KBr): ν (cm^−1^): 2922, 2852, 1458, 1408, 1377, 1215, 1111, 987, 862, 810, 719 (Figure S4).

Trihexyl(tetradecyl)phosphonium heptachlorodiferrat [THTDP][Fe_2_Cl_7_] **10**: [THTDP]Cl (1.5 g, 2.9 mmol) and FeCl_3_·6H_2_O (1.56 g, 5.8 mmol) were stirred at RT for 30 min in a 10 mL round bottom flask. The developing water phase was separated and the ionic liquid was dried for three days under vacuum. [THTDP][Fe_2_Cl_7_] **10** was obtained as yellow oil (2.45 g, 2.9 mmol, 100%). IR: ν (cm^−1^): 2922, 2852, 1458, 1408, 1377, 1216, 1111, 987, 862, 811, 719 (Figure S5).

Trihexyl(tetradecyl)phosphonium chloride with 1.5 eq. FeCl_3_ **11**: [THTDP]Cl (3 g, 5.8 mmol) and FeCl_3_·6H_2_O (2.35 g, 8.7 mmol) were stirred at RT for 30 min in a 10 mL round bottom flask. The developing water phase was separated and the ionic liquid was dried for three days under vacuum. The IL was obtained as yellow oil (4.41 g, 5.8 mmol, 100%). IR: ν (cm^−1^): 2922, 2852, 1457, 1408, 1378, 1215, 1111, 987, 862, 811, 718 (Figure S6).

The spectral data of the ionic liquids were consistent with literature [54,55,56,57] values.

Bis-trihexyl(tetradecyl)phosphonium tetrachlorocuprate [THTDP]_2_[CuCl_4_] **12**: [THTDP]Cl (6 g, 11.6 mmol) and CuCl_2_ (0.78 g, 5.8 mmol) were stirred at RT for 30 min in a 10 mL round bottom flask. The developing water phase was separated and the ionic liquid was dried for three days under vacuum. [THTDP]_2_[CuCl_4_] **12** was obtained as viscous red-brown oil (6.78 g, 5.8 mmol, 100%). IR (KBr): ν (cm^−1^): 2922, 2852, 1458, 1408, 1377, 1215, 1111, 987, 862, 810, 719 (Figure S7).

Trihexyl(tetradecyl)phosphonium trichlorocuprate [THTDP][CuCl_3_] **13**: [THTDP]Cl (3 g, 5.8 mmol) and CuCl_2_ (0.78 g, 5.8 mmol) were stirred at RT for 30 min in a 10 mL round bottom flask. The developing water phase was separated and the ionic liquid was dried for three days under vacuum. [THTDP][CuCl_3_] **13** was obtained as viscous red-brown oil (3.59 g, 5.8 mmol, 100%). IR: ν (cm^−1^): 2922, 2852, 1458, 1407, 1378, 1215, 1111, 986, 862, 720 (Figure S8).

## 5. Conclusions

In the presented work the influence of ionic liquids on an iron trichloride–catalyzed oxidative tandem and hydroarylation reaction of benzaldehyde **1**, aniline **2** and phenylacetylene **3** was investigated. It was possible to use an iron-containing IL as a catalyst and achieve higher yields compared to iron(III) chloride hexahydrate. In addition, it was shown that the [FeCl_4_]^−^ species were not able to catalyze the reaction. The reaction could be performed under a normal atmosphere. The iron(III) functions as a Lewis acid and an oxidant. Air is a co-oxidant and regenerates the iron(II) species to iron(III). The studies will allow the development of further reactions with metalate-based ionic liquids as catalysts.

## Figures and Tables

**Table 1:** Reaction of benzaldehyde **1**, aniline **2** and phenylacetylene **3** to 2,4-phenylquinoline **4**. 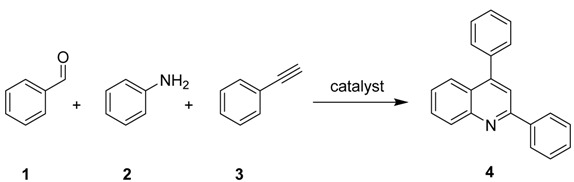

Entry ^a^	Catalyst	Solvents	Time	T [°C]	Yield [%] *^b^*
1	FeCl_3_·6H_2_O	toluene	24 h	110	53
2	[BMIM][FeCl_4_] **5**	toluene	24 h	110	-
3	[TBP][FeCl_4_] **7**	toluene	24 h	110	-
4	[THTDP][FeCl_4_] **9**	toluene	24 h	110	-
5	[BMIM][Fe_2_Cl_7_] **6**	toluene	24 h	110	44
6	[BMIM][Fe_2_Cl_7_] **6**	toluene	18 h	110	40
7	[BMIM][Fe_2_Cl_7_] **6**	-	24 h	130	16
8	[TBP][Fe_2_Cl_7_] **8**	toluene	24 h	110	62
9	[THTDP][Fe_2_Cl_7_] **10**	toluene	24 h	110	54
10	[THTDP][Fe_2_Cl_7_] **10**	1,2-dichloroethane	24 h	85	50
11	[THTDP][Fe_2_Cl_7_] **10**	-	24 h	110	30
12	[THTDP][Fe_2_Cl_7_] **10**	-	24 h	130	38
13 *^c^*	[THTDP][Fe_2_Cl_7_] **10**	-	10 min	110	41
14	[THTDP]Cl/1.5 FeCl_3_ **11**	toluene	24 h	110	48
15	[THTDP]Cl/1.5 FeCl_3_ **11**	1,2-dichloroethane	24 h	85	38
16	[THTDP]Cl/1.5 FeCl_3_ **11**	toluene	14 h	110	38
17	[THTDP]Cl/1.5 FeCl_3_ **11**	-	24 h	110	36
18	[THTDP]Cl/1.5 FeCl_3_ **11**	-	24 h	130	31
19 *^c^*	[THTDP]Cl/1.5 FeCl_3_ **11**	-	20 min	110	43
20 *^c^*	[THTDP]Cl/1.5 FeCl_3_ **11**	-	10 min	110	10
21 *^c^*	[THTDP]Cl **14**	-	20 min	110	-
22	CuCl_2_	toluene	24 h	110	-
23	[THTDP]_2_[CuCl_4_] **12**	toluene	24 h	110	-
24	[THTDP][CuCl_3_] **13**	toluene	24 h	110	-

^a^ General reaction conditions: benzaldehyde **1** (106 mg, 1 mmol), aniline **2** (98 mg, 1.05 mmol), phenyl acetylene **3** (153 mg, 1.5 mmol), catalyst (0.1 mmol), solvent (1 mL) under an atmosphere of air; *^b^* Isolated yields; *^c^* Reaction was performed in a microwave oven at 150 W.

## Supplementary Materials

Supplementary materials can be found at http://www.mdpi.com/1422-0067/17/6/860/s1.
