# Supplementary material for: Influence of Ionic Liquids on an Iron(III) Catalyzed Three-Component Coupling/Hydroarylation/Dehydrogenation Tandem Reaction
Source: Int J Mol Sci. 2016 Jun 1;17(6):860. doi: 10.3390/ijms17060860 (PMC4926394; doi:10.3390/ijms17060860)
Supplement: Supplementary file 1 [file ijms-17-00860-s001.pdf]

Supplementary Materials: Influence of Ionic Liquids on an Iron(III) Catalyzed Three-Component Coupling/Hydroarylation/Dehydrogenation Tandem Reaction

Maren Muntzeck and René Wilhelm

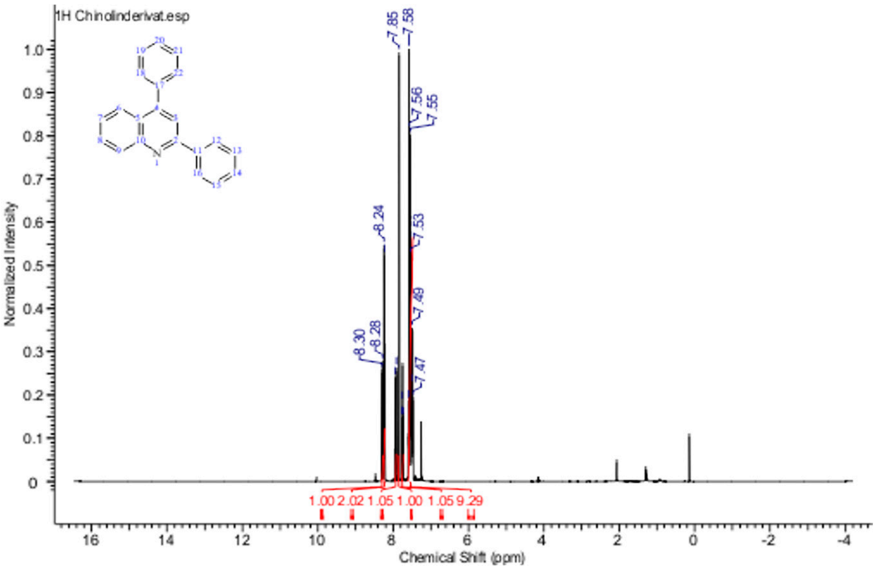

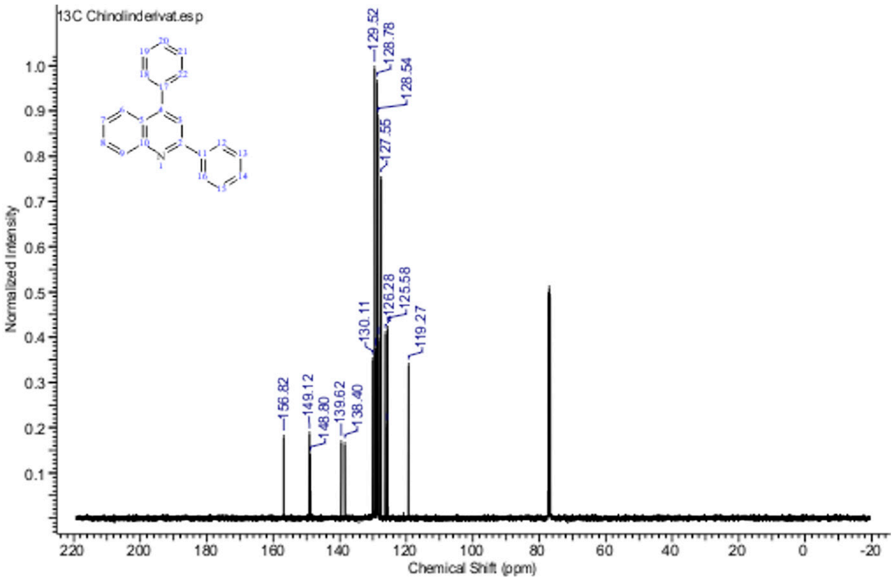

Figure S1. ^{1}H NMR and ^{13}C NMR spectra of 2,4-diphenylquinoline.

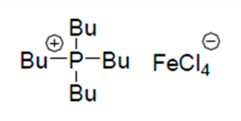

7

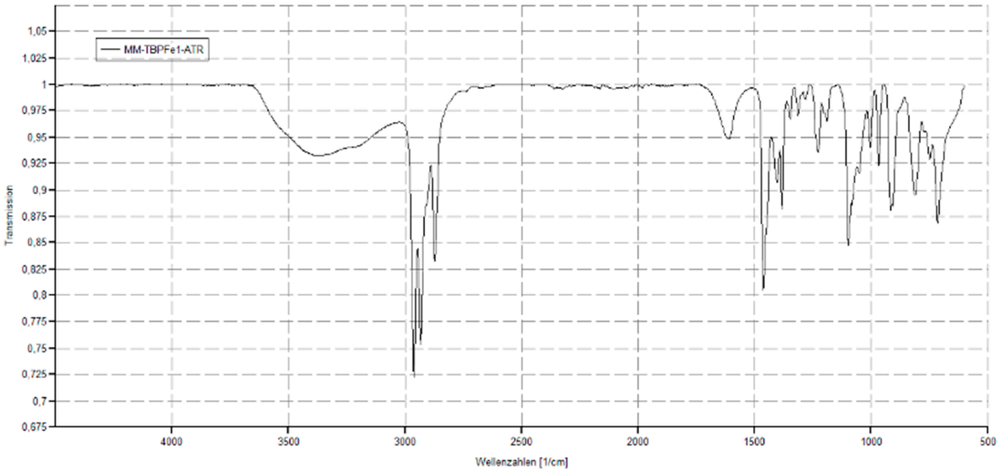

Figure S2. IR-spectrum of [TBP][$FeCl_4$].

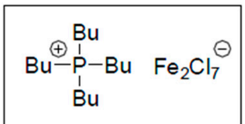

8

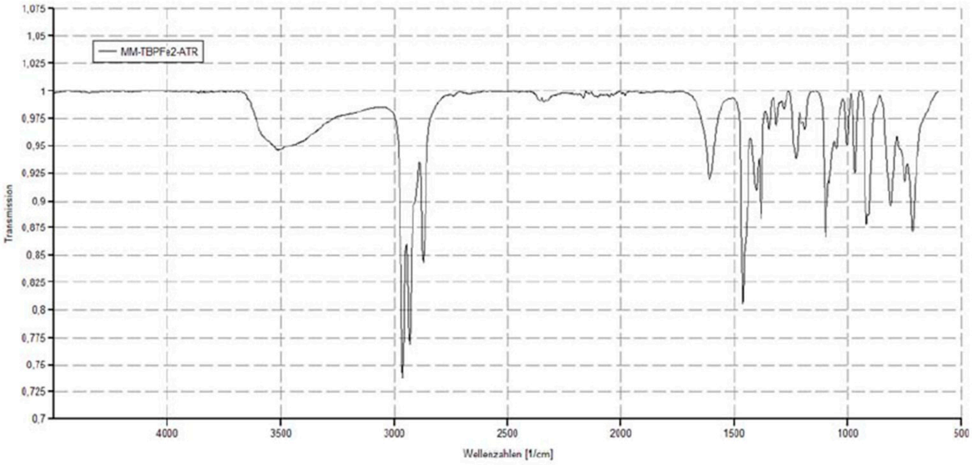

Figure S3. IR-spectrum of [TBP][Fe_2Cl_7].

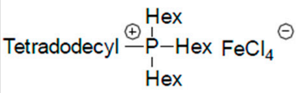

9

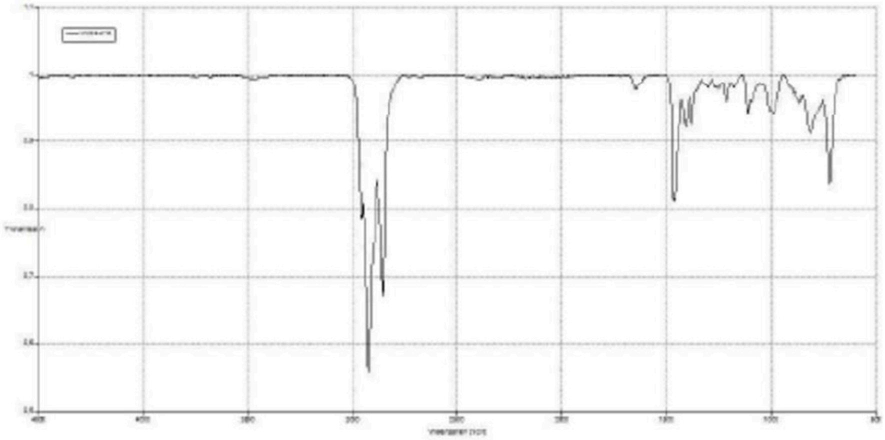

Figure S4. IR-spectrum of [THTDP][$FeCl_4$].

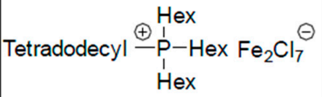

10

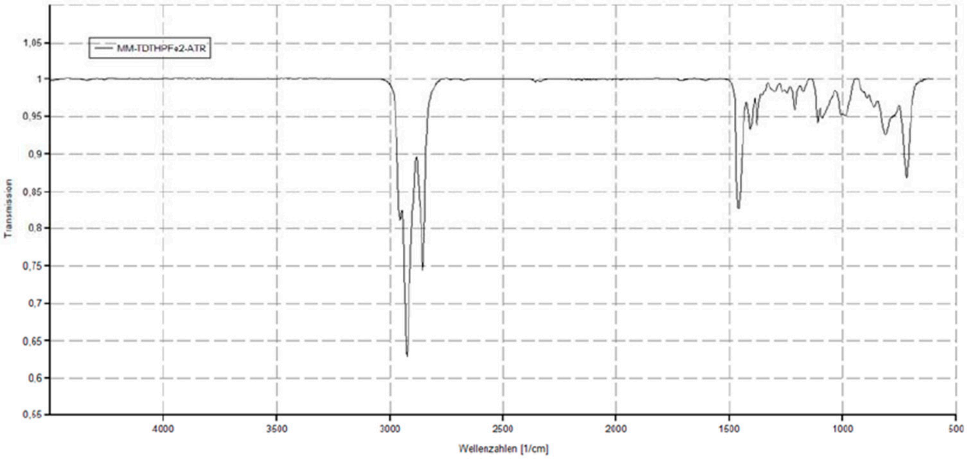

Figure S5. IR-spectrum of [THTDP][Fe_2Cl_7].

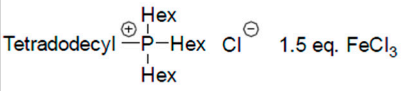

11

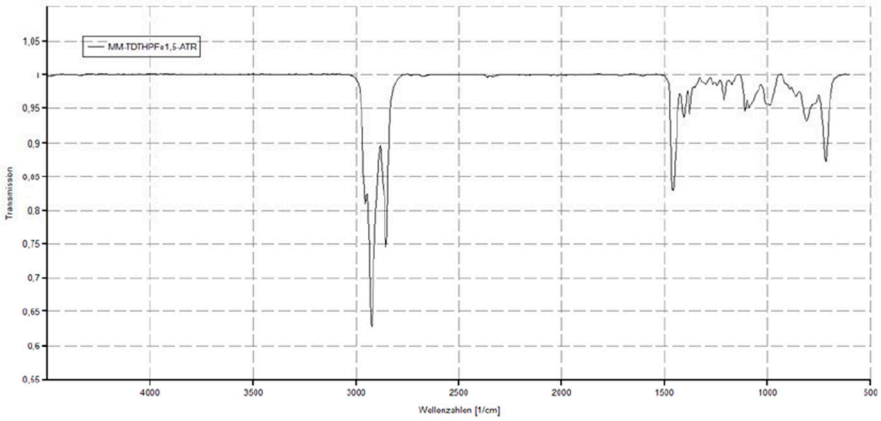

Figure S6. IR-spectrum of [THTDP]Cl/1.5 $FeCl_3$.

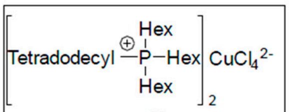

12

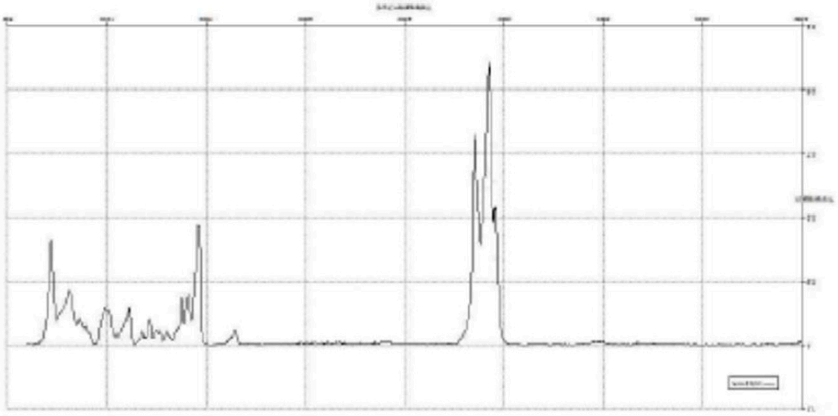

Figure S7. IR-spectrum of $[THTDP]_2[CuCl_4]$.

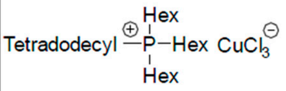

13

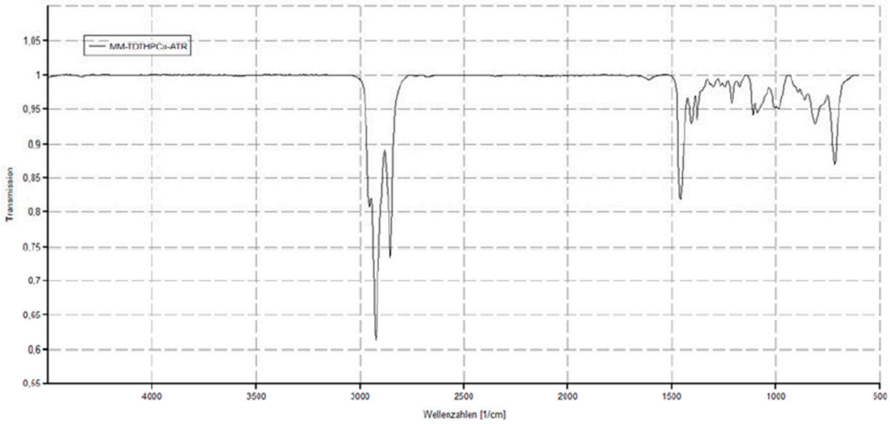

Figure S8. IR-spectrum of [THTDP][$CuCl_3$].
